# Ultrafast Microwave-Assisted Synthesis of Porous NiCo Layered Double Hydroxide Nanospheres for High-Performance Supercapacitors

**DOI:** 10.3390/molecules29112546

**Published:** 2024-05-28

**Authors:** Xing Yang, Qing He, Longbo Hu, Wanglong Wang, Wenmiao Chen, Xing Fang, Jun Liu

**Affiliations:** 1 Key Laboratory of Air-Driven Equipment Technology of Zhejiang Province, Quzhou University, Quzhou 324000, China; yang7092481@163.com (X.Y.); helinqi@163.com (Q.H.); 18283229650@163.com (L.H.); 2 Department of Mechanical Engineering, Zhejiang University of Technology, Hangzhou 310058, China; 221122020226@zjut.edu.cn (W.W.); 15714178548@163.com (W.C.)

**Keywords:** microwave, NiCo-LDH, supercapacitor, ethylene glycol

## Abstract

Currently, new clean energy storage technology must be effective, affordable, and ecologically friendly so as to meet the diverse and sustainable needs of the energy supply. In this work, NiCo-LDH containing intercalated EG was successfully prepared within 210 s using an ultrafast microwave radiation technique. Subsequently, a series of characterization and systematic electrochemical tests were conducted to analyze the composition, structure, and energy storage mechanism of the NiCo-LDH material. The Ni:Co ratio of 5:5 results in the highest capacitance value of 2156 F/g at 1 A/g and an outstanding rate performance of 86.8% capacity retention rate at 10 A/g. The results demonstrated that the unique porous structure of NiCo-LDH and large layer spacing were conducive to more electrochemical reactions. Additionally, an electrochemical test was carried out on the NiCo-LDH as a hybrid supercapacitor electrode material, with NiCo-LDH-5:5 serving as the positive electrode and activated carbon as the negative electrode, the asymmetric supercapacitor can achieve a maximum energy density of 82.5 Wh kg^−1^ and power density of 8000 W kg^−1^. The NiCo-LDH-5:5//AC hybrid supercapacitors own 81.5% cycle stability and 100% coulombic efficiency after 6000 cycles at 10 A/g.

## 1. Introduction

The explosive development of contemporary science and technology, along with the expanding global need for energy and the usage and exploitation of traditional fossil fuels, has resulted in not only a scarcity of energy but also a worsening of environmental pollution and climate change [1,2,3]. Therefore, it is essential to create fresh, efficient, clean, and sustainable energy storage technologies in light of the current situation. Supercapacitors are a new type of energy storage device that can be used in new energy vehicles, smart grids, and other fields. They offer high power density, fast charge and discharge, and extended cycle life, among other advantages [4,5,6]. The way supercapacitors store energy could be divided into two categories: The adsorption mechanism between the electrode and the electrolyte is the basis for energy storage technology known as double-layer capacitors (EDLCs). In electrochemical pseudocapacitors (EPCs), the charge is stored not only in the double electric layer but also through a series of redox reactions of the electrode materials. This allows Faraday capacitors to store more charge than double-layer capacitors and thus have a greater energy density [7,8,9]. According to numerous reports, the performance of supercapacitors largely depends on the properties of the active electrode material, including its composition and structure. The challenge lies in determining which composition, structure, and morphology materials will be used to optimize electrochemical performance, as well as how to efficiently produce these materials while keeping costs low to ensure their application in various fields [10,11,12,13].

NiCo-LDH has attracted much attention among many electrode materials due to its exceptional electrochemical capabilities and distinctive structure. As a type of nanomaterial with an adaptable structure, NiCo-LDH not only has a high specific surface area and fast ion diffusion performance but also combines the advantages of nickel and cobalt, showing high redox activity and electron conductivity. These characteristics provide NiCo-LDH considerable advantages when used as an electrode material in energy storage devices such as supercapacitors [14,15,16,17]. Specifically, Hou et al. [18] successfully created a self-assembled sea urchin-shaped NiCo-LDH using a one-step hydrothermal process. The specific capacitance can reach 808.40 C/g at a current density of 1 A/g and 609.98 C/g at a current density of 10 A/g. Additionally, Zhang et al. [19] synthesized NiCo-LDH hollow spheres using co-glycerate as the cobalt source and as a sacrificial template. This type of hollow sphere assembly, made of NiCo-LDH, has shown great potential in revealing more active areas and enabling faster electrolyte ion diffusion. The unique structural properties of these hollow spheres contribute to their high specific capacitance of 1962 F/g at 1 A/g.

Nevertheless, despite all of the potential benefits, there are still certain obstacles that need to be resolved in practical applications with NiCo-LDH electrode materials. For example, the synthesis method, structural regulation, and performance optimization of NiCo-LDH still need further study and improvement [20,21,22,23]. Therefore, numerous studies have been conducted in an effort to enhance its electrochemical properties. For instance, Han et al. [24] changed the morphology of NiCo-LDH by adjusting the content of polyvinyl pyrrolidone (PVP). When the content of PVP was 1 g, NiCo-LDH transformed into a nanoflower structure, and the electrode exhibited the highest capacitance of 729.4 C/g at 1 A/g. Sun et al. [25] improved the capacitance characteristics of NiCo-LDH by altering the central metal ions. It was observed that when the Ni/Co ratio changed from 1:1 to 1:4, the structure of NiCo-LDHs changed from nanosheets to nanospheres, and finally evolved into nanorods, which exhibited a specific capacitance of 1030 F/g at 3 A/g. Additionally, the introduction of larger interlayer anions or macromolecules was found to be beneficial for improving electrochemical properties. Wang et al. [26] introduced ethylene glycol molecules into the NiCo-LDH interlayer to prepare an electrode with a three-dimensional spongy structure. The electrode demonstrated an exceptionally high specific capacitance of 4160 F/g at 1 A/g due to its enlarged layer spacing, advantageous pore structure, and high specific surface area.

In recent years, microwave radiation technology has emerged as a novel method with a distinct heating mechanism and high energy transfer efficiency. It enables rapid attainment of the required temperature, thereby enhancing reaction rate and product purity [27,28,29,30]. This study presents the swift synthesis of NiCo-LDH with glycol intercalation using microwave-assisted technology, along with further exploration of the impact of the Ni:Co ratio on the morphology and electrochemical characteristics of NiCo-LDH. As an electrode material for supercapacitors, the prepared NiCo-LDH demonstrates exceptional electrochemical properties, including a high specific capacity of 2156 F/g at 1 A/g and an outstanding rate performance with an 86.8% capacity retention rate at 10 A/g. Furthermore, the assembled asymmetric supercapacitor, utilizing activated carbon (AC) as the negative electrode and optimized NiCo-LDH as the positive electrode, demonstrates an outstanding cycling performance by retaining 81.5% of its capacity after 6000 cycles even at a high current density of 10 A/g. Additionally, it achieves a high energy density of 82.5 Wh kg^−1^ at a power density of 800 W kg^−1^.

## 2. Results and Discussion

### 2.1. Characterization

**Figure 1:**
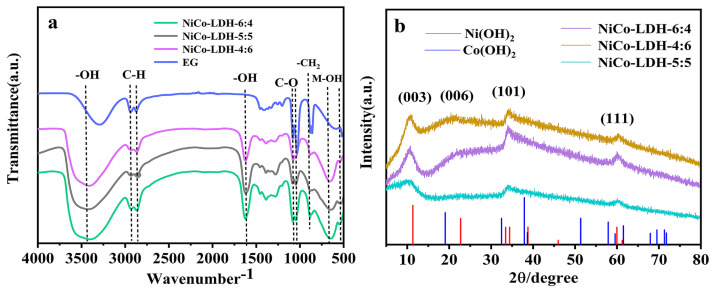
(**a**) FT-IR spectra and (**b**) XRD patterns of NiCo-LDH-4:6, NiCo-LDH-5:5, and NiCo-LDH-6:4.

Fourier infrared spectroscopy (FT-IR, Figure 1a) of all the samples reveals a prominent and broad adsorption band near 3411 cm^−1^, corresponding to the stretching vibration of the -OH group involved in hydrogen bonding. The strong infrared absorption peak at 1624 cm^−1^ is attributed to the O-H bending vibration from interlayer H_2_O, indicating the presence of crystalline H_2_O in the NiCo-LDH intercalation structure. Additionally, characteristic absorption peaks of EG are clearly observed in all samples, including antisymmetric and symmetric stretching vibrations corresponding to C-H bonds at 2927 cm^−1^ and 2870 cm^−1^, as well as stretching and bending vibration absorption peaks corresponding to C-O at 1085 cm^−1^ and 1046 cm^−1^, respectively. The rocking vibration peaks of CH_2_ are located at 883 cm^−1^ and 862 cm^−1^. The wide absorption band is near 3284 cm^−1^, which belongs to the -OH group of EG. The shift of the -OH absorption peak reflects the change in the EG environment to some extent, which may be attributed to the formation of new hydrogen bonds between the NiCo-LDH nucleus and hydroxyl group on the EG molecule. The presence of the aforementioned absorption peaks provides evidence that EG has been effectively intercalated into NiCo-LDH. Additionally, the bands at 650 cm^−1^ and 550 cm^−1^ are attributed to the bending and stretching vibrations of M(Ni/Co)-OH. These findings suggest the successful preparation of NiCo-LDH with EG intercalation [31,32].

Figure 1b depicts the X-ray diffraction (XRD) analysis of the crystal structure of the NiCo-LDH materials. The predominant angle reflections at 9.5°, 22.5°, 34.3°, and 60.3° correspond to the (003), (006), (101), and (111) crystal faces, indicating the presence of a-phase Ni(OH)_2_ and a-phase Co(OH)_2_ in NiCo-LDH [33]. Among them, the peaks located at 9.5°, 22.5°, and 34.3° correspond to the (003), (006), and (101) crystals of Ni(OH)_2_ (PDF# 38-0715). The peak at 60.3° belongs to the crystal plane of Co(OH)_2_ (PDF# 30-0443). Additionally, the Bragg equation was utilized to calculate the interlayer spacing of the (003) crystal faces, revealing a significantly larger interlamellar distance of approximately 8.6 Å compared to the published value of 7.8 Å for NiCo-LDH [34], which is primarily attributed to the introduction of macromolecule EG. This larger interlayer distance is beneficial for storing more electrolytes and facilitating faster ion transfer between layers, thereby enhancing its electrochemical performance. Especially when the Ni/CO ratio is 5:5, the NiCo-LDH material gradually exhibits a broad and weak diffraction peak, indicating reduced crystallinity. The relatively weak crystallinity facilitates the penetration of electrolyte ions to some extent [35].

**Figure 2:**
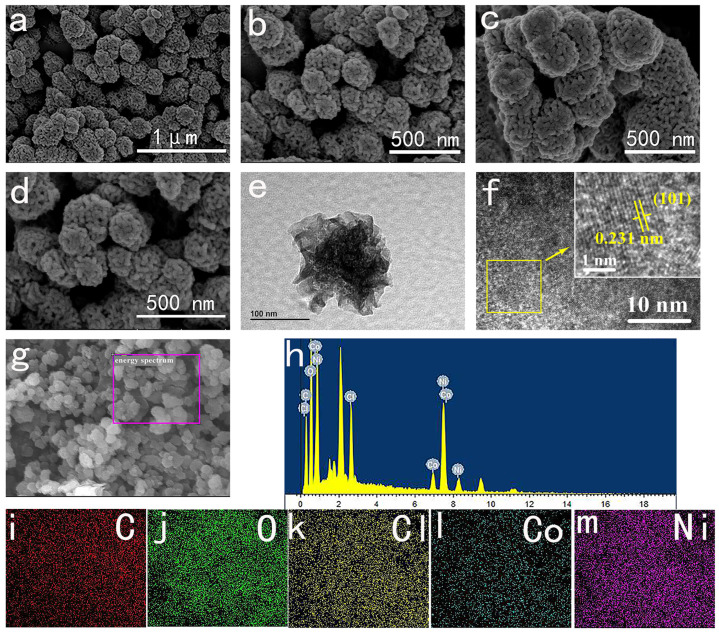
(**a**,**b**) SEM images of NiCo-LDH-5:5; (**c**,**d**) SEM of NiCo-LDH-6:4 and NiCo-LDH-4:6; (**e**,**f**) TEM images of NiCo-LDH-5:5; (**g**,**h**) EDS of NiCo-LDH-5:5; (**i**–**m**) element mapping of NiCo-LDH-5:5.

The morphology of NiCo-LDH was examined using scanning electron microscopy (SEM) and transmission electron microscopy (TEM). As depicted in Figure 2a, it is evident that NiCo-LDH-5:5 consists of porous nanospheres with an average size ranging from 100 to 300 nm. Figure 2b–d display the SEM images of NiCo-LDH materials with varying Ni/Co ratios at a scale of 500 nm. It is apparent that the NiCo-LDH-5:5 samples exhibit more uniform and smaller particle-size nanospheres. In the case of NiCo-LDH-5:5, TEM images in Figure 2e reveal that the nanosphere is comprised of numerous nanoparticle clusters with abundant interconnecting pores, facilitating increased electrolyte contact with the active substance and enhancing exposure to electrochemically active sites. The high-resolution TEM images in Figure 2f reveal an interplanar spacing of 0.23 nm, corresponding to the (101) plane of NiCo-LDH. The EDS spectrum depicted in Figure 2h–m demonstrates even dispersion of C, O, Ni, Co, and Cl elements throughout the material, further confirming the presence of Cl^−^ as an intercalation anion in NiCo-LDH. Combined with the FT-IR and XRD analysis above, these results confirm the successful preparation of NiCo-LDH with an intercalation structure, wherein Cl^−^, H_2_O, and EG molecules are successfully inserted into the material.

**Figure 3:**
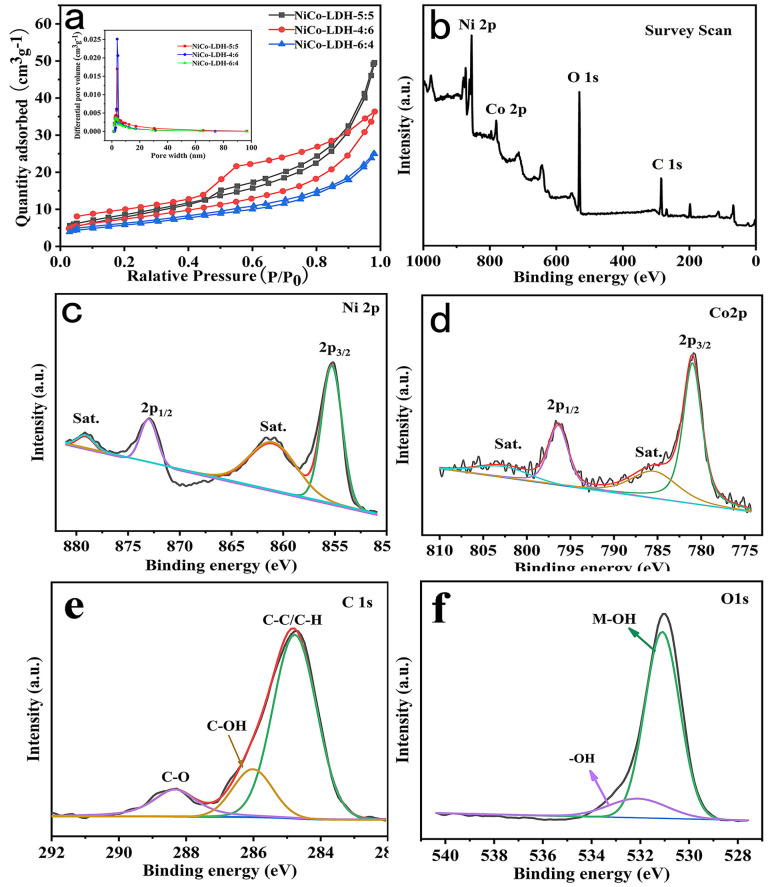
(**a**) N_2_ adsorption–desorption isotherms of NiCo-LDH; (**b**–**f**) XPS survey spectra of NiCo-LDH-5:5.

N_2_ adsorption–desorption isotherms were utilized to investigate the structure properties of NiCo-LDH. As shown in Figure 3a, the isotherms of all samples match to an H3 type, which is a typical feature of slitlike holes formed by the aggregation of particles [24]. According to the Brunauer–Emmett–Teller (BET) method, the specific surface areas of NiCo-LDH-5:5, NiCo-LDH-4:6, and NiCo-LDH-6:4 were 38.35 m^2^g^−1^, 36.79 m^2^g^−1^, and 20.79 m^2^g^−1^, respectively, with corresponding pore volumes of 0.080 cm^3^g^−1^, 0.055 cm^3^g^−1^ and 0.037 cm^3^g^−1^, respectively. Notably, NiCo-LDH-5:5 exhibited the highest specific surface area and pore volume among the samples studied. It can be clearly observed from the BJH pore size distribution curves in Figure 3a that the pore size of NiCo-LDH materials ranges from 1 to 100 nm, most of which is centered at 4 nm. Based on the pore size distribution results, it can be inferred that NiCo-LDH materials are predominantly comprised of mesoporous structures. These structures facilitate electrolyte entry and offer ample ion adsorption sites, thereby leading to outstanding electrochemical properties [35].

To investigate the fundamental composition and chemical bonding state of the NiCo-LDH in further detail, XPS tests were performed on NiCo-LDH-5:5, and the results are displayed in Figure 3. The survey spectrum (Figure 3b) indicated the presence of Ni, Co, C, and O elements in the NiCo-LDH sample, and the elemental composition represents the fundamental property of NiCo-LDH. In the Ni 2p spectrum of Figure 3c, the two distinct characteristic peaks (855.3 eV and 873.0 eV) and two satellite peaks (861.2 eV and 879.1 eV) can be clearly observed. The two characteristic peaks of 855.2 eV and 873.0 eV correspond to the spin orbits of Ni 2p_3/2_ and Ni 2p_1/2_, respectively, with a spin energy interval of 17.7 eV indicating the presence of the Ni^2+^ state [21,31]. Likewise, the two main spin–orbit peaks observed in the Co 2p spectra (Figure 3d) at 780.9 eV and 796.4 eV are attributed to Co 2p_3/2_ and Co 2p_1/2_, respectively. These peaks are accompanied by two satellite peaks at 802.7 eV and 785.6 eV, indicating the coexistence of both Co^2+^ and Co^3+^ ions in NiCo-LDH [36,37,38]. The C 1s spectrum of NiCo-LDH clearly shows three peaks of ethylene glycol, which are located at 284.4 eV (C-C/C-H), 286.1 eV (C-OH), and 288.3 eV (C-O) [39]. The spectrum of O 1s can be clearly seen, and the binding energy for O 1 s was related to M-OH and -OH bonds at 531.1 eV and 532.3 eV [20]. This result is consistent with the infrared spectra, indicating the successful preparation of NiCo-LDH.

### 2.2. Electrochemical Properties

**Figure 4:**
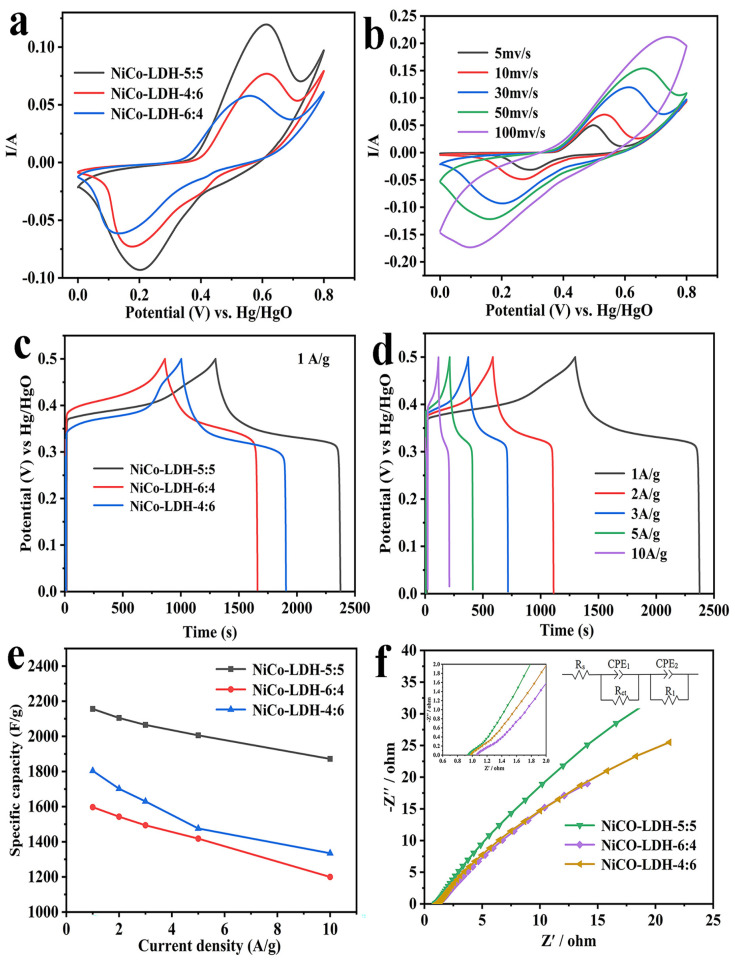
(**a**) Comparison of CV curves at 30 mV/s; (**b**) CV curves of NiCo-LDH-5:5 with varied scan rates; (**c**) GCD curves of NiCo-LDH-5:5, NiCo-LDH-4:6, and NiCo-LDH-6:4 at 1 A/g; (**d**) GCD curves of NiCo-LDH-5:5 at different current densities; (**e**) specific capacitance of NiCo-LDH-5:5, NiCo-LDH-4:6, and NiCo-LDH-6:4 at different current densities; (**f**) Nyquist plots of NiCo-LDH-5:5, NiCo-LDH-4:6, and NiCo-LDH-6:4.

The electrochemical properties of all NiCo-LDH electrodes were researched by a three-electrode test system with a 2 M KOH aqueous solution. Figure 4a exhibits a typical CV curve for all electrodes within the voltage range of 0–0.8 V and at a scan rate of 30 mV/s. It can be observed that all samples exhibit a pair of strong redox peaks, specifically, oxidation peaks between 0.55 and 0.65 V, and reduction peaks between 0.1 and 0.2 V, which are mainly due to the reversible redox reaction of (Ni^2+^/Ni^3+^, Co^2+^/Co^3+^). The corresponding faradic reaction process is as follows [40]:

(1) $$\mathrm{Ni}(\mathrm{OH}{)}_{2}+OH^{-}↔\mathrm{NiOOH}+H_{2}O+e^{-}$$

(2) $$\mathrm{Co}(\mathrm{OH}{)}_{2}+OH^{-}↔\mathrm{CoOOH}+H_{2}O+e^{-}$$

(3) $$\mathrm{CoOOH}+OH^{-}↔\mathrm{Co}O_{2}+H_{2}O+e^{-}$$

The NiCo-LDH-5:5 electrode exhibited the largest CV curve area and the highest peak current value among all samples, indicating its superior suitability for charge storage. This could be a result of the largest specific surface area of NiCo-LDH-5:5, which exposes more surface atoms or ions, thus increasing the number of active sites for electrochemical reactions. Figure 4b depicts the CV curve of NiCo-LDH-5:5 at different scanning rates. It is noted that the shape of the CV curve hardly changes with the increase in the scanning rate, suggesting that it has preeminent electrochemical reversibility. Meanwhile, the anode peak and cathode peak on the curve move in the direction of the positive and negative potential, respectively, as the scanning rate increases, which is mainly due to an increased electrode polarization at high scanning rates.

The galvanostatic charge–discharge (GCD) tests were conducted on all electrodes within a potential range of 0 to 0.5 V. Figure 4c illustrates the GCD curve for all electrodes at a current density of 1 A/g. Among them, the NiCo-LDH-5:5 electrode exhibited the longest discharge time and highest specific capacity, while, conversely, the NiCo-LDH-6:4 electrode demonstrated the shortest discharge time, consistent with the results of CV testing. Furthermore, the NiCo-LDH-5:5 electrode was chosen for additional analysis. Figure 4d illustrates its GCD curves at various current densities, all of which exhibit satisfactory symmetry, indicating high coulomb efficiency and excellent electrochemical reversibility. Subsequently, based on the GCD test results, the specific capacities of all electrodes under different current densities were calculated and summarized in Figure 4e. The NiCo-LDH-5:5 electrode demonstrated the highest specific capacity, measuring 2156 F/g, 2105 F/g, 2065 F/g, 2006 F/g, and 1872 F/g at current densities ranging from 1 to 10 A/g, respectively. In contrast, the NiCo-LDH-6:4 electrode exhibited the lowest specific capacity. Additionally, the capacity retention rate of the NiCo-LDH-5:5 electrode (86.8% at 10 A/g) was significantly higher than that of both the NiCo-LDH-4:6 electrode (74% at 10 A/g) and NiCo-LDH-6:4 electrode (75.1% at 10 A/g). The comparison of the NiCo-LDH with other studies of NiCo-LDH and its composites is shown in Table 1. The NiCo-LDH in this work has exceeded many previously reported results.

Obviously, the electrode material of NiCo-LDH-5:5 has a higher specific capacity and relatively excellent rate capability. The excellent electrochemical performance can be attributed to the following: (1) The porous structure between the cluster nanoparticle, which facilitates electrolyte entry, increases the effective contact area with the active substance, exposes more electrochemically active sites, and ultimately enhances the specific capacity of the electrode. In addition, a large number of mesoporous pores on the nanospheres are conducive to faster penetration of the electrolyte and effectively shorten the ion diffusion distance, and it can ensure that more electrochemical reactions occur at high current densities, thus improving the rate capability of the electrodes. (2) The unique intercalated structure, which includes EG, H_2_O, and Cl^−^ in the electrode material, effectively expands the interlayer spacing. This contributes to an improved contact between electrolyte ions and the active site, effectively shortens the diffusion path of ions/electrons, and significantly enhances the specific capacity and rate performance. (3) The synergistic effect of nickel–cobalt bimetallics significantly enhances the electrochemical performance of the electrode. During the electrochemical reaction, a portion of Co(OH)_2_ undergoes permanent conversion to the highly conductive CoOOH, thereby improving the overall electronic conductivity of the electrode.

The electrodes were subjected to EIS testing to analyze their conductivity and ion diffusion behavior. The EIS test was conducted within a frequency range of 0.01 kHz to 100 kHz, revealing a sloped line in the high-frequency range and a semicircle in the low-frequency range. Specifically, the slope of the line is associated with electrolyte ion diffusion, while the width of the semicircle represents the Faraday charge transfer impedance (Rct), and the intercept on the real axis indicates internal resistance (Rs). The ZView2.1c software was used to fit the EIS results, and the equivalent circuit for fitting the EIS curve is shown in Figure 4f. The specific values of Rs and Rct are shown in Table 2. The results indicate that, compared to other electrodes, the NiCO-LDH-5:5 electrode exhibits smaller Rs and Rct values, as well as a low-frequency linear region closer to vertical orientation, suggesting faster rates of ion transport. This is mostly because of the large specific surface area and unique porous structure of the NiCo-LDH-5:5 electrode. For porous materials, their internal pore structure may facilitate the transport of electrons and ions. Therefore, a larger surface area may improve the electrical conductivity and ion transport performance of the electrode material, thereby reducing the electrochemical impedance.

By assembling a NiCo-LDH//AC hybrid supercapacitor (HSC) with NiCo-LDH-5:5 as the positive electrode and activated carbon (AC) as the negative electrode, the performance of the NiCo-LDH//AC in electrochemical energy storage devices was investigated in a 2 M KOH electrolyte. According to the hybrid capacitor energy storage mechanism, both the positive and negative electrodes have distinct mechanisms for storing energy during the electrochemical reaction process. The positive electrode utilizes a redox reaction process to consume anions (OH^−^) and store energy, while the negative electrode stores and releases charge through the adsorption and desorption of cations (K^+^).

**Figure 5:**
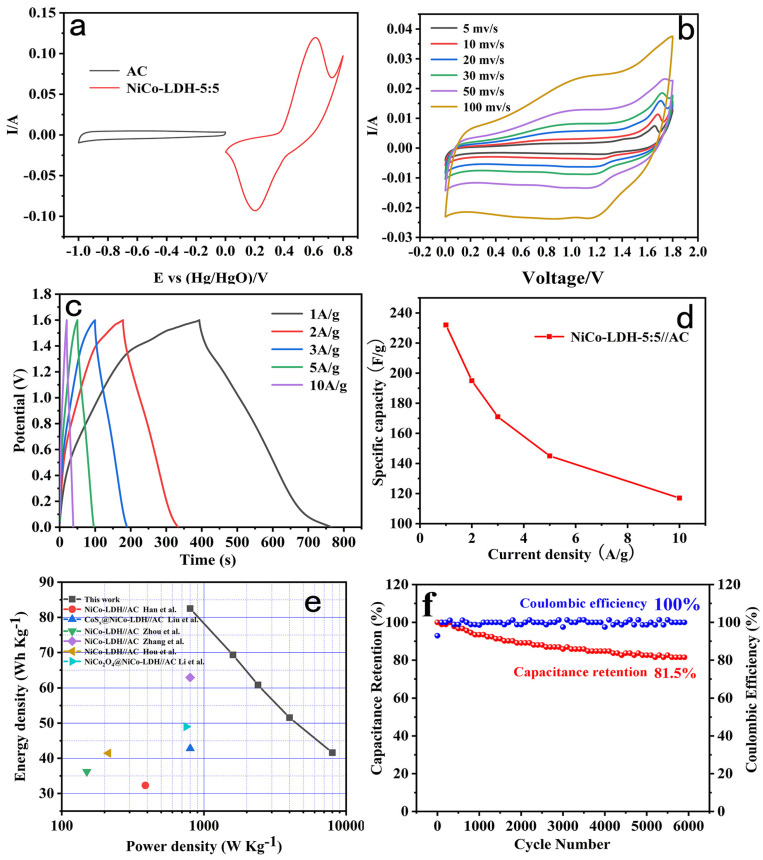
(**a**) CV curves of AC and NiCo-LDH-5:5 electrodes at a scan rate of 30 mV s^−1^ in a three-electrode system; (**b**) CV curves of the NiCo-LDH-5:5//AC at the different scan rates; (**c**) GCD curves and (**d**) the specific capacitance of the NiCo-LDH-5:5//AC at different current densities; (**e**) Comparison of power and energy densities; (**f**) Cycling stability and Coulombic efficiency of the asymmetric supercapacitor.

Figure 5a shows the CV curve of positive electrodes (NiCo-LDH-5:5) and negative electrodes (AC) with a scanning rate of 30 mV/s. It is evident that the positive electrode of NiCo-LDH-5:5, which is a material similar to a battery type, has a pair of significant redox peaks on its CV curve, whereas the negative electrode AC, which is a typical capacitor-type material, has a practically rectangular CV curve. Upon combining these materials into the HSC, the potential window could be enlarged to 1.8 V and the curve displays the inherent characteristics of positive and negative materials. In practical terms, a high stability working voltage exceeding 1.5 V generally meets the requirements for commercial applications.

The shape of the CV curve in Figure 5b has no discernible change as the scan rate increases, demonstrating its rapid charging and discharging capabilities. Furthermore, the GCD curve of the HSC in Figure 5c exhibits excellent symmetry at various current densities (1~10 A/g), indicating optimal electrochemical reversibility. This means that the supercapacitor can maintain its performance consistently under different operating conditions, ensuring reliable and stable energy storage. The specific capacitance calculation results provided in Figure 5d show that the HSC device using the NiCo-LDH-5:5//AC demonstrated an impressive capacitance of 232 F/g at 1 A/g, which was maintained at 118 F/g even at a high current density of 10 A/g. These results highlight the exceptional energy storage capability of this supercapacitor, making it a promising candidate for practical use in electronic devices or renewable energy systems.

The values of energy density and power density are crucial parameters for evaluating the performance of energy storage devices. The values of energy density and power density are obtained by calculating the GCD curve with the following formula [40]:

(4) $$E=\frac{C\times {\left(\Delta V\right)}^{2}}{7.2}$$

(5) $$P=\frac{3600\times E}{\Delta t}$$

where the energy density (Wh kg^−1^) is denoted by *E*, while *C* represents the specific capacitance of the HSC (F/g). Δ*V* represents the discharge potential window (*V*), *P* is the power density (W kg^−1^), and Δ*t* is the discharge time (s).

The corresponding energy and power density of NiCo-LDH-5:5//AC are shown in Figure 5e; when the power density is 8000 W kg^−1^, the energy density of the HSC is 41.6 Wh kg^−1^, while when the energy density is 82.5 Wh kg^−1^, the power density remains at 800 W kg^−1^. This outcome is better than several recent reports of electrochemical energy storage devices constructed of nickel–cobalt compounds [18,19,24,32,41,42], indicating that it has great potential in practical applications. Furthermore, the cyclic stability of an energy storage device is a critical factor. To evaluate this aspect, 6000 cycles were conducted at a current density of 10 A/g, and the corresponding graphs are shown in Figure 5f. The specific capacitance ultimately maintained at 81.5% of the initial value after 6000 cycles; meanwhile, NiCo-LDH-5:5 revealed less variation in coulombic efficiency with cycling. The coulomb efficiency of the NiCo-LDH-5:5 electrode reached 100% after activation from the initial 92.9%, and still maintained 100% coulomb efficiency after 6000 cycles, demonstrating exceptional cycling stability and high charge storage and release efficiency of the NiCo-LDH//AC HSC device for long-term use.

## 3. Materials and Methods

### 3.1. Materials

Nickel chloride hexahydrate (NiCl_2_·6H_2_O), Cobalt chloride hexahydrate (CoCl_2_·6H_2_O), and N-N-Dimethyformamide (DMF) were acquired from MACKLIN chemical reagent Co., Ltd. (Shanghai, China). Taicang Hu Test Reagent Co., Ltd. (Suzhou, China) as the supplier of potassium hydroxide and ethylene glycol (EG), while Tian Jin Chemical Technology Co., Tianjin, China, provided the polyvinylidene fluoride (PVDF) and acetylene black. Before being used, none of the aforementioned products underwent any extra processing.

### 3.2. Synthesis of NiCo-LDH

**Figure 6:**
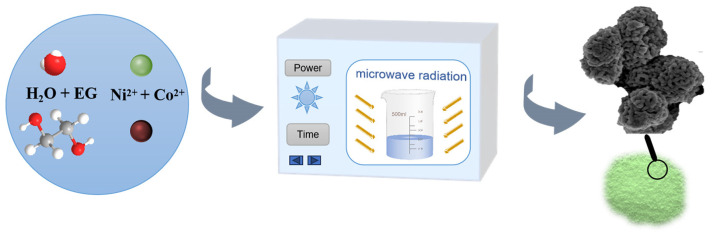
Synthesis of NiCo-LDH.

The porous NiCo-LDH nanosphere was synthesized using a highly efficient microwave radiation method, as depicted in Figure 6. The detailed preparation process is outlined as follows: Solvent A was prepared by mixing 100 mL of N-N-Dimethyformamide with 100 mL of ethylene glycol. Subsequently, 1.14 g of CoCl_2_·6H_2_O and 1.14 g of NiCl_2_·6H_2_O were dissolved in 200 mL of solvent A, which was then agitated for one hour to form a homogeneous solution B. Solution B was then transferred to a PANASONIC NN-GF352 M, 2450 MHz microwave oven and heated for 210 s at 600 W to initiate the chemical reaction. Upon termination of the reaction, a blue suspension appeared, which was filtered and washed alternately with deionized water and DMF until the filtrate became colorless. Finally, the filtered green product was transferred to a vacuum oven and dried at 80℃ for 12 h to obtain NiCo-LDH. Following this, NiCo-LDH samples with varying Ni:Co ratios (4:6, 5:5, and 6:4) were synthesized and designated as NiCo-LDH-4:6, NiCo-LDH-5:5, and NiCo-LDH-6:4, respectively.

### 3.3. Preparation of NiCo-LDH Electrodes

Nickel foam (1 × 1 cm^2^), acetylene black, and PVDF were, respectively, used as a collector fluid, conductive agent, and binder for working electrodes. First, the electrode material (NiCo-LDH), PVDF, and acetylene black were thoroughly ground and mixed according to the mass ratio of 1:1:8 until forming a uniform black slurry, and then the slurry was evenly coated with nickel foam and dried at 80 °C for 24 h. It is worth noting that effective electrodes with a load of around 0.8–1.2 mg/cm^2^ of the electrode material were selected, and finally, these effective electrodes were pressed into working electrodes by the tablet press.

### 3.4. Characterization

The structure analysis of NiCo-LDH materials mainly depends on an X-ray diffraction (XRD) analyzer (Rigaku ULTIMAIV, Akishima, Japan) to determine the crystal structure. Scanning electron microscopy (SEM, Hitachi-SU-8100, Dongjing, Japan) and transmission electron microscopy (TEM, FEI TECNAI G2 F30, Hillsboro, OR, USA) can be used to observe the morphology, particle size, and other microscopic characteristics of NiCo-LDH. The chemical composition of NiCo-LDH materials was analyzed by Energy dispersive X-ray spectroscopy (EDS, IXRF3310, Austin, TX, USA), Fourier transform infrared spectroscopy (FT-IR, THERMO FISHER NICOLET 6700, Waltham, MA, USA), and X-ray photoelectron spectroscopy (XPS, ESCALAB 250XI, Waltham, MA, USA). These technologies can accurately determine the element content, functional groups, and other information about the elements in the material. In addition, the specific surface area and pore size distribution of the material can be calculated using the Brunauer–Emmett–Teller method (BET, AUTOSORB IQ, Boynton, FL, USA).

The electrochemical performance of the NiCo-LDH was assessed utilizing an electrochemical workstation (CHI660E, Shanghai, China) in a 2M KOH electrolyte. In a three-electrode system, NiCo-LDH electrodes were used as working electrodes, while platinum electrodes and Hg/HgO electrodes served as counter and reference electrodes, respectively. The range of scans for the cyclic voltammetry (CV) testing was 0 to 0.8 V. Under various current densities from 1 A/g to 10 A/g, the galvanostatic charge–discharge (GCD) tests were performed in the voltage range of 0 to 0.5 V. The frequency range covered by the electrochemical impedance spectroscopy (EIS) experiments was 100 kHz–1 Hz.

## 4. Conclusions

In summary, the porous nanosphere morphology of NiCo-LDH was effectively synthesized in 210 s using an ultrafast microwave-assisted method. The cluster nanoparticles were self-assembled into porous nanospheres with a diameter of 100–300 nm. Simultaneously, intercalated molecules such as ethylene glycol were introduced to NiCO-LDH to further expand the layer spacing. The unique structure is conducive to the electrode to store more electrolyte ions and accelerate the ion transport, and thus NiCO-LDH shows excellent electrochemical performance. Among them, the NiCo-LDH-5:5 electrode exhibited outstanding comprehensive performance, demonstrating a high specific capacity of 2156 F/g at 1 A/g. Moreover, the NiCo-LDH-5:5//AC hybrid supercapacitors exhibit exceptional energy and power density as well as outstanding long-cycle stability (81.5% capacity retention rate after 6000 cycles). This work offers a unique way for the development of superior electrode materials for energy storage and an efficient synthesis process.

## Figures and Tables

**Table 1:** Capacitance of NiCo-LDH materials.

Sample	Electrolyte	Specific Capacitance	Ref.
NiCo-LDH hollow spheres	6 M KOH	1962 F g^−1^ at 1 A g^−1^	[19]
NiCo-LDH/Co_3_S_4_	6 M KOH	728.1 C^−1^ at 1 A g^−1^	[20]
NiCo-LDH	6 M KOH	724.9 C g^−1^ at 1 A g^−1^	[24]
3D flower-on-sheet nanostructure of NiCo LDHs	6 M KOH	1187.2 F g^−1^ at 1 A g^−1^	[32]
PPy@NiCo(OH)_2_	6 M KOH	1469.25 F g^−1^ at 1A g^−1^	[37]
NiCo-LDH NFs@Co(OH)_2_ nanosheets	2 M KOH	858.9 F g^−1^ at 0.5 A g^−1^	[38]
core-shell hollow CoSx@NiCo-LDH	3 M KOH	680.8 C g^−1^ at 1 A g^−1^	[41]
Porous NiCO-LDH nanospheres	2 M KOH	2156 F g^−1^ at 1 A g^−1^	This work

**Table 2:** Rs and Rct values of fitting circuit.

	NiCO-LDH-4:6	NiCO-LDH-5:5	NiCO-LDH-6:4
Rs (Ω)	0.99	0.964	1.07
Rct (Ω)	0.203	0.138	0.283

## Data Availability

The data that support the findings of this work are available from the corresponding author upon reasonable request.
